# Impact of surgical approach on development of surgical-site infection following internal mammary-artery, coronary-artery bypass graft procedures

**DOI:** 10.1017/ice.2023.88

**Published:** 2023-11

**Authors:** Polly H. van den Berg, Baevin Fesser, Venkatachalam Senthilnathan, Kamal R. Khabbaz, Sharon B Wright

**Affiliations:** 1 Division of Infectious Diseases, Pennsylvania Hospital, Philadelphia, Pennsylvania; 2 Division of Infection Prevention, Beth Israel Lahey Health, Cambridge, Massachusetts; 3 Division of Cardiac Surgery, Beth Israel Deaconess Medical Center, Boston, Massachusetts; 4 Division of Infectious Diseases, Beth Israel Deaconess Medical Center, Boston, Massachusetts

Deep incisional and organ-space surgical-site infections (SSIs) are infrequent but serious complications of coronary artery bypass graft (CABG) procedures. Arterial revascularization using internal mammary artery (IMA) grafts is associated with improved cardiac outcomes.^1^ The use of bilateral IMA (BIMA) grafting redirects sternal blood flow to the heart and may increase SSI risk due to lower sternal tissue perfusion. A skeletonized approach to IMA vessel harvest, wherein the IMA is dissected from surrounding tissue preserving collateral sternal blood flow, may decrease SSI risk compared with a pedicled approach, in which the IMA is mobilized within a tissue pedicle. It remains unclear whether the use of BIMA grafting compared with the use of a single IMA (SIMA) is an independent risk factor for SSI. It is also uncertain whether surgical approach to graft harvest, skeletonized versus pedicled, affects SSI development. Conflicting results have been reported on the impact of BIMA grafting and harvest technique on development of SSI.^2–5^ In this study, we described the incidence of post-CABG SSI and assessed potential patient and procedural SSI risk factors, including IMA number and harvest technique.

## Methods

We conducted a retrospective cohort study of consecutive adult patients who underwent a CABG procedure with at least 1 IMA graft at an academic tertiary-care center between July 2017 and June 2020, identified through the institution’s Society of Thoracic Surgeons database maintained by the Division of Cardiac Surgery. Data were electronically abstracted from hospital data records including demographics, comorbidities, graft number, surgical approach, surgeon, discharge location, and microbiological culture reports. Deep incisional and organ-space SSIs post-CABG within 90 days of procedure date were identified by infection preventionists using standard National Healthcare Safety Network definitions as part of routine surveillance. We calculated the incidence of post-CABG deep incisional and organ-space SSI. Bivariate analyses were performed using the Fisher exact test to identify potential patient and procedural risk factors for SSI, including surgical approach. Statistical analyses were performed using SAS version 9.4 software (SAS Institute, Cary, NC). All reported *P* values are 2-sided. This study was deemed exempt from review by the institutional review board.

## Results

Overall, 1,591 CABG procedures with at least 1 IMA graft were performed; 550, 561, and 480 procedures were performed in each respective year of the study period. Furthermore, 1,244 CABGs (78.2%) were performed with a single IMA and 347 (21.8%) were performed using BIMA. In terms of surgical technique, 322 (92.8%) of BIMA CABGs were skeletonized versus 219 (17.6%) of SIMA CABGs. The baseline patient risk factors (Table 1) did not differ between IMA groups, except the SIMA group was more likely to meet criteria for extreme obesity (BMI ≥ 40 kg/m^2^; 5.3% vs 2.0%; *P* = .008).

**Table 1.** Potential Risk Factors for Deep Incisional and Organ-Space Post-CABG SSI

Variable	SSI (n=19), No. (%)	No SSI (n=1,533), No. (%)	*P* Value
**Patient risk factors**			
Diabetes	17 (89.5)	726 (47.4)	.0002
Sex, female	11 (57.9)	305 (19.9)	.0003
Discharge to rehabilitation facility	14 (77.8)	564 (36.8)	.0007
Age ≥75 y	9 (60.0)	388 (26.4)	.0065
Extreme obesity (BMI ≥ 40 kg/m^2^)	4 (21.1)	63 (4.1)	.008
White	15 (79.0)	1066 (69.5)	.50
Ever smoker	7 (36.8)	641 (41.8)	.82
**Procedural risk factors**			
BIMA graft	6 (31.6)	334 (21.8)	.28
Pedicled harvest technique	14 (73.7)	1009 (65.8)	.62

Note. CABG, coronary artery bypass graft; SSI, surgical-site infection; BIMA, bilateral internal mammary artery.

Overall, 19 deep incisional and organ-space SSIs occurred during the study period. The overall post-CABG SSI incidence rate was 1.2 per 100 procedures, with 1.0 and 1.7 SSIs per 100 procedures following SIMA and BIMA approaches, respectively. Over the 3-year study period, the proportion of CABG procedures using SIMA grafting increased (from 77.5% to 76.1% to 81.5%) and skeletonized grafting increased (from 29.1% to 34.2% to 39.4%). Over the same period, overall SSI incidence decreased (from 1.8 to 1.1 to 0.6 SSI per 100 procedures). *Staphylococcus* spp caused 8 infections (ie, 3 coagulase-negative staphylococci, 3 methicillin-resistant *S. aureus*, 2 methicillin-susceptible *S. aureus*) and *Pseudomonas aeruginosa* caused 4.

Diabetes, female sex, discharge to a rehabilitation facility, age ≥75 years, and BMI ≥40 kg/m^2^ (ie, extreme obesity) were significant predictors of post-CABG SSI on bivariate analysis (Table 1).

## Discussion

In our study, the incidence of deep incisional and organ-space SSI following CABG with at least 1 IMA was 1.2%, which is similar to the range of 1%–4% reported in the literature.^2–8^

Potential patient risk factors for SSI were diabetes,^3,4,6–8^ female sex,^3,4,7,8^ older age,^6^ and obesity,^3,4,6–8^ matching findings from other studies. In addition, we found that discharge to a rehabilitation facility was associated with post-CABG SSI, which is likely explained by confounding patient comorbidities requiring discharge to a facility instead of home.

In terms of procedural risk factors, use of a BIMA graft, compared with use of a SIMA graft, and surgical graft harvest technique (pedicled vs skeletonized) were not significant predictors of SSI (Table 1). We observed an increase in the use of SIMA and skeletonized grafts over time, which appeared to be accompanied by a decrease in SSI incidence. Notably, no other practice changes were implemented during this period.

This study had several limitations. It had a retrospective single-center design. Given that the number of deep incisional and organ-space SSIs was low, we had insufficient power to detect differences between groups. Another limitation of this study was that surgeon preference and perception of patient risk for infection likely influence method of revascularization, which may have affected surgical outcome and SSI development. BIMA utilization and IMA vessel skeletonization are associated with longer operative time, which may have affected surgical approach.

This study provides preliminary data on the impact of surgical technique to reduce deep incisional and organ-space SSIs following CABG procedures utilizing at least 1 IMA. The overall incidence of SSI following BIMA-skeletonized CABGs was similar compared to SIMA-pedicled CABGs (1.6% vs 1.3%), suggesting that BIMA-skeletonized CABGs may be performed safely. Because surgical approach and number of IMA vessels are potentially modifiable factors, even in patients with more traditional risk factors for SSI, additional research is needed to validate the significance of these findings.
